# Optimization of Polydimethylsiloxane-Modified Composite Synthesis and Its Impact on Collagen Interactions: Perspectives for Biomedical Applications

**DOI:** 10.3390/ma17051045

**Published:** 2024-02-24

**Authors:** Leszek Kadziński, Bogdan Banecki

**Affiliations:** Intercollegiate Faculty of Biotechnology of UG and MUG, University of Gdańsk, Abrahama Str. 58, 80-307 Gdansk, Poland

**Keywords:** silica composite, polydimethylsiloxane, collagen, synthesis optimization

## Abstract

This research explores how silica composites modified with polydimethylsiloxane interact with collagen, aiming to enhance their application in the biomedical field. By adjusting the amount of polydimethylsiloxane in these composites, we evaluated their capacity to bind with collagen, an essential feature for biomaterials used in tissue engineering and drug delivery. Our findings reveal that incorporating polydimethylsiloxane into silica composites significantly boosts collagen attachment, indicating strong binding interactions. Notably, the collagen adhered to the composites maintains its natural structure, ensuring its functionality and compatibility with living tissues. This aspect is critical for biomaterials that support cell growth and regeneration in tissue scaffolds. Additionally, this study investigates how the viscosity of polydimethylsiloxane influences collagen binding, offering insights into the tailoring of composite properties for better biological performance. This work highlights the potential of polydimethylsiloxane-modified silica composites in creating innovative biomaterials for regenerative medicine and targeted therapeutic delivery.

## 1. Introduction

Bones comprise a heterogeneous matrix including mineral and organic phases alongside water. This composition varies with age, gender, anatomical location, and health, crucially influencing bones’ mechanical and biological functions. The mineral phase predominates, with the organic phase mainly consisting of type I collagen, constituting about 90% of bones’ protein content. The bone matrix is further enriched with a variety of proteins [1].

The cellular architecture of bones encompasses osteogenic precursor cells, osteoblasts, osteoclasts, osteocytes, and hematopoietic elements. Osteoblasts synthesize the extracellular matrix, chiefly type I collagen, while osteoclasts engage in bone resorption. Osteoblasts mature into osteocytes during mineralization, and the vascular nature of bones is essential for cellular functionality and activation [2].

Bones’ regenerative capacity involves the inflammatory response, new bone formation, and remodeling post injury. The process begins with hematoma formation following vascular disruption, serving as a scaffold for inflammatory mediators and cells. This phase leads to the recruitment and differentiation of cells essential for bone regeneration [3].

Inadequate natural regeneration necessitates bone grafting, employing autografts, allografts, or synthetic substitutes. Bone graft substitutes aim to provide osteogenic, osteoinductive, osteoconductive, and osteointegrative properties, employing various materials and methodologies [4,5]. This study focuses on bioactive porous composites potentially interacting with inflammatory or bone cells via collagen, bypassing cross-linking stages.

Collagen, primarily type I, is integral in bones, forming fibrils with triple-helical structures. Despite its biocompatibility and tensile strength, collagen’s mechanical limitations are addressed by integrating it with non-collagenous materials like silica composites or bioglass to improve mechanical properties. The mechanical robustness and structural integrity of collagen scaffolds diminish upon exposure to water, constraining their utility in specific tissue applications. Enhancing the scaffold’s mechanical attributes can be accomplished through intermolecular cross-linking via either physical or chemical techniques. Furthermore, amalgamating collagen with additional substances, including both natural and synthetic polymers as well as inorganic compounds, is a common strategy to bolster the mechanical strength of these scaffolds [6]. Silica composites, recognized for their high porosity and surface area, are typically produced via sol–gel methods, yielding biocompatible structures suitable for drug delivery and controlled-release applications [7], often modified with compounds such as polydimethylsiloxanes (PDMS) [8].

Hydroxylated PDMS of various chain lengths has been utilized to modify silica composites, affecting drug release from xerogels [8,9,10,11]. PDMS modification is favored due to its non-toxic, biocompatible, and hydrophobic properties. Previous studies have demonstrated PDMS’s ability to interact with collagen, with the interaction strength being inversely proportional to the chain length and sufficiently robust to bind collagen without altering its structure or function [12].

A vital challenge in silica composite research is establishing an efficient synthesis protocol, a task complicated by the vast array of potential variations. These variations encompass a range of parameters, including precursor concentration, water to precursor molar ratios, modifier presence and concentration, catalyst type and concentration, and the drying conditions prescribed by the sol–gel process. The design of experiments methodology serves as an invaluable tool in this context, offering a means to assess the influence of individual factors on the synthesis process and elucidate parameter interactions that are not discernible through single-factor analyses. Notable among such experimental designs are the Box–Behnken Design and the Central Composite Design. The traditional univariate approach, which adjusts a single factor while holding others constant, falls short of addressing the complexity of the synthesis process. Conversely, a full factorial design demands an impractically high number of experimental runs. The compromise proposed by George E. P. Box and Donald Behnken in 1960, through their introduction of a three-factorial design, significantly mitigates this issue by reducing the requisite number of experiments [13,14]. A distinctive feature of the Box–Behnken Design is its exclusion of extreme-level variable combinations, thereby streamlining the experimental process without compromising the comprehensiveness of the analysis.

This study aims to assess the influence of PDMS-modified silica composites on collagen interaction, striving to design biomaterials integrating the optimal properties of each component. Understanding the interactions between hydrophobic proteins and intracorporeal silicone polymers will enhance the specificity and regenerative efficacy of biomaterials, exploiting protein adsorption to silicone polymers as a beneficial phenomenon.

## 2. Materials and Methods

### 2.1. Materials and Scheme of the Research Methodology

In each experiment, type I collagen from calf and human skin (Sigma-Aldrich, Poznań, Poland) was utilized. Tetramethoxysilane (TMOS, 98%) and hydroxy-terminated polydimethylsiloxanes (PDMSs) with viscosities of 25, 65, and 750 cS were procured from Sigma-Aldrich, Poznań, Poland. The “Sircol Collagen Assay” (Biocolor, County Antrim, UK) was employed for determining the native collagen concentration. For quantifying the total collagen concentration via the Lowry method, Folin–Ciocalteu’s phenol reagent (Sigma-Aldrich, Poznań, Poland) was utilized. Post-fluorescence labeling collagen purification was conducted using a Sephadex G-25 Medium column (GE Healthcare, Warsaw, Poland). Unless specified otherwise, additional reagents were obtained from Sigma-Aldrich (Poznań, Poland).

**Figure 1:**
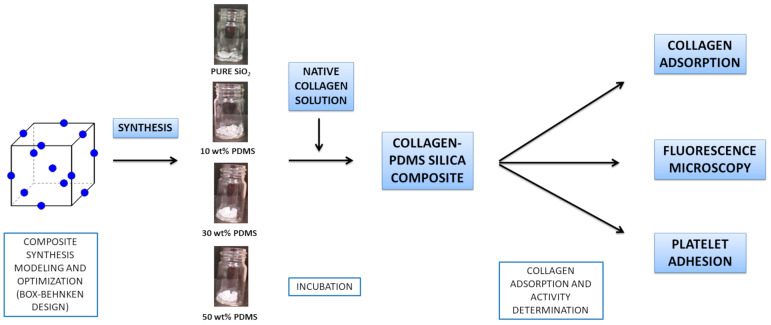
Conceptual scheme of the research methodology.

The project entailed several phases, schematically illustrated in Figure 1. Initially, the process involved the modeling and optimization of the synthesis parameters for silica-based composites. Subsequently, composites with varying PDMS contents were produced, alongside a control sample of pure silica composites. At this stage, the hydroxylated form of PDMS was employed, facilitating the integration of this polymer into the silica composite structure. The next phase involved incubating each composite with a native collagen solution to investigate potential interactions between the protein and either silica or PDMS. This was assessed by monitoring collagen concentration changes in the incubation solution. Given the observed interactions, post-incubation composites were further analyzed to detect adsorbed protein and evaluate its biological activity. Additionally, the effect of PDMS chain length on collagen interaction was examined, and collagen was fluorescently labeled to visualize its location on the composite.

### 2.2. Preparation of Native Collagen Solution

Approximately 1 mg of collagen was transferred into a 1 mL Eppendorf tube. Subsequently, 0.5 mL of 0.1% acetic acid aqueous solution was added. The mixture was then incubated at 4 °C with gentle agitation overnight. Following incubation, the concentration of the native collagen fraction was determined as outlined in Section 2.6. The solution was subsequently diluted to a final concentration of 0.2 mg/mL using 0.1% acetic acid and maintained at 4 °C for additional analysis.

### 2.3. Modeling and Optimization of Silica Composites Synthesis

The analysis of the effects of PDMS content (% *w*/*w*), buffer pH, and H_2_O:TMOS molar ratio on the gelation time was performed using a Box–Behnken Design (BBD) with a response surface methodology (RSM). It is a set of methods that allows one to minimize the number of necessary experimental runs and optimize processes more efficiently in terms of costs and labor by fitting the mathematical model and determining a regression equation containing linear and quadratic effects and interactions between variables. The graphic representation of such a regression equation is called response surface. The levels of independent variables are shown in Table 1. The relationship of the preparation parameters (independent variables) and gelation time (the response) was fitted to a predictive second order polynomial equation:

(1) $$Y_{i}=\beta_{0}+\sum_{i=1}^{n}\beta_{i}X_{i}+\sum_{i=1}^{n}\beta_{ii}X_{i}^{2}+\sum_{i=1}^{n-1}\sum_{j=i+1}^{n}\beta_{ij}X_{i}X_{j}$$

where *Y_i_* is the predicted response (gelation time); subscripts *i* and *j* have values from 1 to the number of variables; *β*_0_ is a constant; *β_i_* is a linear coefficient; *β_ii_* is the quadratic coefficient; *β_ij_* is the cross-product coefficient; *n* is the number of factors; and *X_i_* and *X_j_* are the coded dimensionless values of the analyzed variables.

The software Design-Expert 13 (Stat-Ease, Minneapolis, MN, USA) was used for the experimental design, the analysis of variance (ANOVA), and the graphical analysis of the data. The statistical significance of the model was expressed by an *F*-test, and the quality of its fit was evaluated by the coefficient of determination *R*^2^. The significance of the regression coefficients was tested by means of Student’s *t*-test, and the *p* values were used to determine the significance of each coefficient.

### 2.4. Preparation of Mesoporous Silica Composites

Silica composites were synthesized utilizing a one-step sol–gel process, adhering to a previously optimized methodology established by our research group [15]. In a concise manner, the silica precursor tetramethyl orthosilicate (TMOS) was combined with double-distilled water (ddH_2_O), maintaining a water-to-TMOS molar ratio of 11:1. This mixture was then acidified through the introduction of approximately 100 µL of 0.1 N hydrochloric acid per 15 mL of the final solution volume. Subsequently, the resultant solutions underwent mixing using a magnetic stirrer for 10 min at room temperature (22 °C ± 2), followed by sonication for an additional 10 min at 30 °C. Methanol was then incorporated to achieve a final concentration of 10% (*w*/*v*), and hydroxy-terminated polydimethylsiloxane (PDMS) was added at the desired weight percentage. The solutions underwent further sonication for 30 min at 30 °C with a frequency of 35 kHz. The prepared sols were then integrated with a sodium acetate buffer (50 mM, pH 5.3) and transferred into Petri dishes. Each resulting sol’s volume was measured to be 15 mL. The incorporation of the acetate buffer facilitated the gelation process, subsequent to which the gels were aged overnight at 4 °C. Following the aging process, the gels were dried, ground into a powder, and sorted by particle size by sieving in a sieve cascade. Composite fractions exhibiting particle sizes ranging from 100 to 200 μm were selected for subsequent research applications.

### 2.5. Characterization of Composites by Fourier Transform Infrared Spectroscopy (FTIR)

Attenuated Total Reflection-Fourier Transform Infrared (ATR-FTIR) spectra were recorded with a Perkin-Elmer Spectrum One spectrometer with the UATR accessory (Perkin-Elmer, Waltham, MA, USA). All the spectra were recorded at a resolution of 2 cm^−1^, and a total of 32 scans were accumulated for each spectrum along with the background.

### 2.6. Determination of Native Collagen Concentration

To determine the concentration of native collagen, a commercial kit supplied by Biocolor was employed. Quantification was achieved via a tailored version of the manufacturer’s protocol. In summary, 100 µL of the collagen test solution and 500 µL of Sircol Dye were combined in a 2 mL round-bottom Eppendorf tube and incubated for 30 min at ambient temperature with agitation. The mixture was then centrifuged at 15,000 rcf for 10 min. The supernatant was removed, and the pellet was resuspended in 500 µL of ethanol, followed by vortexing. After a second centrifugation at 15,000 rcf for 2 min, the supernatant was discarded, and the pellet was redissolved in 500 µL of Alkali reagent and incubated in a thermo-mixer for 10 min at room temperature. The resultant solution was subjected to a spectrophotometric analysis at 540 nm. A five-point calibration curve, with triplicate measures at each concentration, was established using external standards ranging from 0.05 to 0.4 mg/mL for accurate concentration determination.

### 2.7. Determination of Total Collagen Concentration

To determine the total collagen concentration, the modified Lowry method was employed [16]. In summary, 50 µL of the collagen solution and a corresponding amount of siloxane, along with 50 µL of acetic acid solution, 90 µL of solution A, and 10 µL of solution B, were combined in a 2 mL Eppendorf tube. This tube was subsequently incubated in a thermo-mixer at 50 °C for 20 min with constant agitation at 500 rpm. Following incubation, the sample was allowed to cool to room temperature before the addition of 300 µL of Folin–Ciocalteu reagent. The sample was then vortexed for approximately 15 s and further incubated in the thermo-mixer at 20 °C for 10 min, maintaining constant agitation at 500 rpm. The resulting solution was subjected to a spectrophotometric analysis at 650 nm. To ascertain the final concentration, a five-point calibration curve was established, with three replicate measurements at each concentration. The calibration employed external standards ranging from 0.01 to 0.3 mg/mL.

### 2.8. Fluorescent Labeling of Collagen Using Fluorescein Isothiocyanate (FITC)

For the fluorescent labeling of collagen, a modified approach based on the Antonio Baici method [17] was employed. In brief, approximately 3.0 mL of native collagen solution was introduced into a dialysis bag. This bag was subsequently immersed in a beaker containing 500 mL of collagen-labeling buffer 1 (comprising 0.25 M sodium bicarbonate, 0.4 M NaCl, pH 9.5) for one hour at 4 °C. This dialysis process was repeated for an additional 24 h and then again for 1 h under identical conditions. Subsequently, 20.0 mg of fluorescein isothiocyanate (FITC) was solubilized in 200 mL of collagen-labeling buffer 1. Thereafter, the dialysis bag containing the collagen was placed into this prepared buffer for 24 h in the absence of light, at 4 °C, with continuous stirring.

To eliminate any unbound fluorescent marker, the dialysis bag was then immersed in 500 mL of collagen-labeling buffer 2, which consists of 0.2% (*v*/*v*) acetic acid at a pH of 4.0, for 1 h, under the same previously mentioned conditions. This dialysis step was conducted twice more for 24 h each, using 500 mL of collagen-labeling buffer 2 for each session. The fluorescently labeled collagen solution was then processed through a Sephadex G-25 column, with elution at a flow rate of 0.2 mL/min. Fractions of 1 mL were collected, and those with the highest protein concentration were transferred to a centrifuge vial. Collagen was precipitated by the gradual addition of NaCl to a final concentration of 5% (*w*/*v*), followed by centrifugation for 1 h at 50,000 rcf and 4 °C. The supernatant was discarded, and the pellet was resuspended in 3.0 mL of collagen-labeling buffer 2 and incubated for 24 h in the absence of light at 4 °C with constant stirring. The final step involved measuring the concentration of the native labeled collagen according to the standard procedure, as outlined in Section 2.4.

### 2.9. Investigation of the Interactions between PDMS-Modified Silica Composites and Collagen

For each instance of protein–composite interaction analysis, the protocol was conducted as follows: 50 mg of the composite was transferred to a round-bottom Eppendorf tube. Subsequently, 1.0 mL of a 0.1% acetic acid aqueous solution was added. The mixture was then incubated at controlled conditions (30 °C) overnight to reach equilibrium. The following day, the tube was gently centrifuged at 200 rcf to sediment the composites. The supernatant was discarded, and 1.0 mL of the native collagen solution was introduced into the incubation mixture, achieving a final protein concentration of 0.2 mg/mL. At specific intervals, aliquots were withdrawn from the incubation mixture to assess the concentration of the native collagen fraction (as outlined in Section 2.6) and the total collagen content (as outlined in Section 2.7).

### 2.10. Platelet Adhesion Determination

In a preparatory step, venous blood was collected from healthy individuals who refrained from aspirin and any pharmacological agents for a minimum of two weeks. Platelet-rich plasma (PRP) was procured through the centrifugation of anticoagulated blood (prepared with 3.8% sodium citrate in a 1:9 *v*/*v* ratio) at 200 × rcf for 10 min at room temperature. Determination of platelets adhesion to test the composites was performed using a lactate dehydrogenase assay kit (TOX7 kit; Sigma-Aldrich, Poznań, Poland). A total of 50 mg of the composite was transferred to a round-bottom Eppendorf tube and was washed three times with 0.1% acetic acid aqueous solution. The composite was immersed in 0.2 mL of fibrillogenesis buffer (60 mM NaH_2_PO_4_, 1.4% NaCl (*w*/*v*), pH 7.5), and 0.2 mL of 0.2 mg/mL collagen solution was added. The solution was incubated for 3 h at 37 °C. The composites were rinsed twice with a PBS buffer (prewarmed to 37 °C) to remove the unbound collagen. A total of 1 mL of PRP was added to the new tubes. Next, the pretreated composites were added and incubated for 90 min at 37 °C. The platelet suspensions were then removed from the tubes, and the test composites were washed six times with a PBS buffer. The composites were transferred to fresh tubes to eliminate determining platelets that had adhered to the tube walls; a 1/10 volume of LDH Assay Lysis Solution was added and incubated at 37 °C for 45 min to lyse the adhered platelets. Equal volumes of platelet lysates (50 μL) and LDH reaction solution (50 μL) were transferred to the wells of a non-adhesive 96-well plate and incubated for 30 min at room temperature. A total of 10 μL of stop solution (1 M HCl in PBS) was then added to the wells, and the absorbance was measured at 490 nm against a 650 nm reference using a microplate reader.

### 2.11. Statistical Analysis

All the experiments were performed in three repetitions. The results are presented as mean ± standard deviation. The statistical analysis was performed using the OriginPro 2022 software (OriginLab, Northampton, MA, USA) by means of ordinary one-way ANOVA followed by a Tukey’s test.

## 3. Results

**Figure 2:**
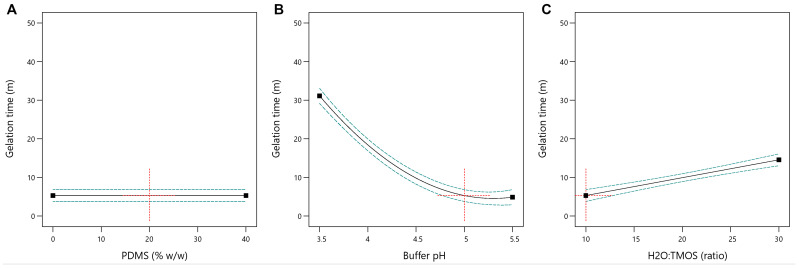
Effect of PDMS content (**A**), buffer pH (**B**), and H_2_O:TMOS molar ratio (**C**) on the sol gelation time (blue dashed line—95% confidence interval; red dotted cross—values of tested variables fixed).

During composite synthesis modeling and optimization, under the experimental conditions tested, the gelation time was in the range of 5–41 min. The statistical analysis of the model showed that it was significant (*p*-value < 0.0001) with a not significant lack of fit. A statistically significant relationship was demonstrated between the molar ratio of water to TMOS and buffer pH (*p*-value < 0.0001)—the higher the ratio (and the lower the buffer pH), the longer the sol-to-gel transition time (Table 2). It was shown that the content of PDMS had no significant impact on this process (Figure 2).

The values of the adjusted and predicted coefficient of determination R^2^ after reduction of the model are high and in good agreement (0.9827 and 0.9010, respectively). The model has a high precision of 49.13, which indicates an adequate signal; therefore, it can be used to predict the response. The equation of the model is the following:

gelation time (min) = 14.44 − 13.12 × B + 4.63 × C + 8.18 × B^2^ (2)

where B is the buffer pH, and C is H_2_O:TMOS (molar ratio).

The mathematical model recovered in the previous steps was used to optimize the synthesis parameters. The goal was to minimize the gelation time. Numerical optimization was performed resulting in a good desirability of fitting (1.00). Using optimized synthesis parameters, three types of composites were obtained with 10, 30, and 50% (*w*/*w*) of PDMS and one type without PDMS added. The observed gelation times were in good agreement with the predicted ones, both for the composites with PDMS (X1, X2, X3) and without PDMS (X4). The optimized synthesis parameters as well as the predicted and determined values of the responses are presented in Table 3.

**Figure 3:**
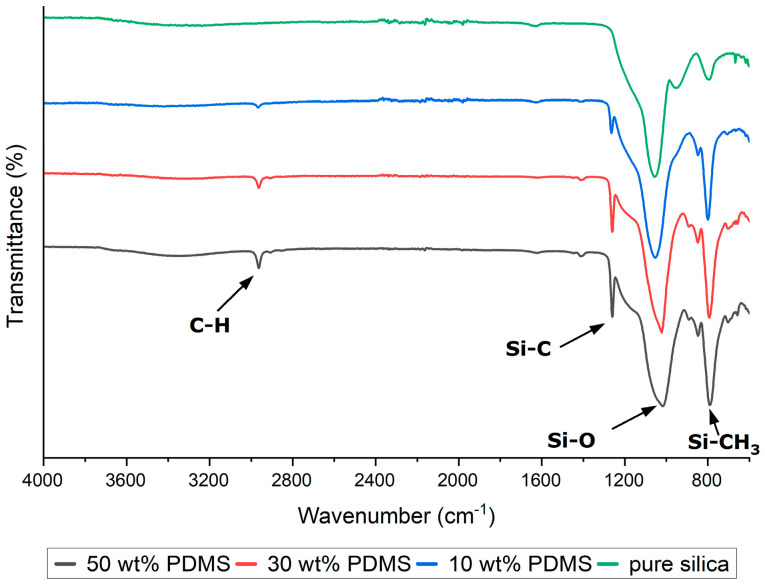
FTIR spectra of PDMS-modified silica composites. All the spectra are normalized by the absorption band of the Si-O vibration at about 1000 cm^−1^.

The synthesized silica-based composites were subjected to ATR-FTIR analysis to confirm the completeness of the hydrolysis of the precursors and the incorporation of PDMS into the silica structure. The PDMS exhibited characteristic infrared peaks at 789–796 cm^−1^, attributed to the rocking motion of the -CH_3_ groups and the stretching of the Si-C bonds in Si-CH_3_; at 1020–1074 cm^−1^, corresponding to the stretching vibrations of Si-O-Si; at 1260–1259 cm^−1^, indicative of CH_3_ deformation in Si-CH_3_; and at 2950–2960 cm^−1^, associated with the asymmetric stretching of the CH_3_ groups in Si-CH_3_ (Figure 3). These spectral features serve as evidence for the successful hydrolysis of precursor materials and the effective embedding of PDMS within the silica matrix, thereby validating the type and content of the modifier (PDMS).

**Figure 4:**
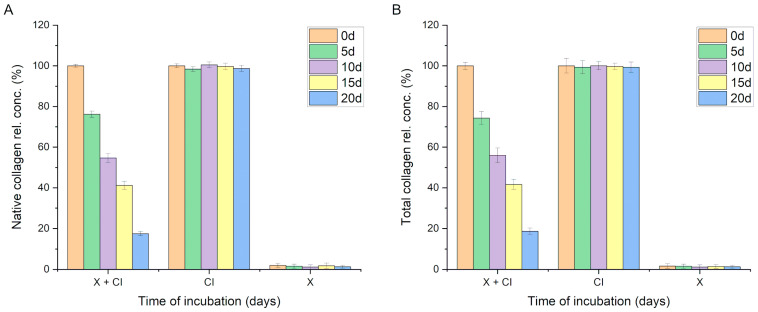
(**A**) Results of the determination of the concentration of native collagen in the solution above the composite during incubation for 0, 5, 10, 15, and 20 days (d = days); (**B**) results of the determination of the concentration of total collagen in the solution above the composite during incubation (X + CI—collagen and PDMS-modified silica composite incubation mixture; CI—control collagen solution; and X—control composite in the buffer).

During the incubation of the native collagen solution (0.2 mg/mL) with the PDMS-modified silica composite, a gradual decrease in the concentration of native collagen in the solution was observed. The incubation was conducted over 20 days following the procedure outlined in Section 2.9. A statistically significant reduction in the concentration of native collagen in the solution was recorded after just 5 days of incubation (from 100% at the start of incubation to 76.2% ± 1.5, *p* < 0.001). Further declines in the native collagen content in the incubation solution were noted at subsequent time points, reaching 17.5% ± 1.0 after 20 days of incubation. No statistically significant decrease in native collagen quantity was observed in control sample 1 (0.2 mg/mL collagen solution without composite). Conversely, control sample 2 contained the same quantity of identical PDMS-modified silica composite but did not include the protein (only a buffer); in this case, no signal indicating collagen detection was observed (Figure 4A).

These results suggest that collagen was either adsorbed onto the composite surfaces or had lost its native structure. In cases of denaturation, the collagen’s spatial structure is altered, which impedes the interaction with the Sirius Red dye (used in the method described in Section 2.6). Consequently, a modified Lowry method was employed for our protein concentration analysis. Upon optimization, this method allows for the efficient determination of collagen concentration and is less sensitive to protein conformational changes, thus enabling the determination of the total collagen concentration (both native and denatured forms). Analyses conducted using this procedure yielded results very similar to those obtained when analyzing the native collagen content, indicating a significant statistical decrease in the collagen content in the solution over time. This phenomenon was not observed in the control collagen solution (no concentration changes over time) nor in the control solution containing a PDMS-modified silica composite in the buffer (signal < method’s limit of detection, Figure 4B).

Collagen denaturation, resulting in the loss of its triple-helical structure, leads to a notable decrease in the measured collagen concentration using Sirius Red staining compared to the modified Lowry method. This is because Sirius Red dye has a reduced affinity for denatured collagen molecules, which lack the intact triple helix necessary for optimal dye binding. The modified Lowry method, which assesses protein content based on the reaction of peptide bonds with copper ions and their subsequent detection by the Folin–Ciocalteu reagent, is less affected by changes in protein conformation. Therefore, it provides higher collagen concentration measurements under conditions where the triple-helical structure is disrupted. Given that the observed collagen concentration levels obtained through both selected methods were closely comparable, the hypothesis suggesting protein denaturation in the solution was dismissed. The alternative hypothesis concerning the interaction of collagen with the PDMS-modified silica composite was investigated through two approaches. Initially, a modified method involving Sirius Red dye was employed. Modifications included the centrifugation of the sample containing the collagen solution and the PDMS-modified silica composite, followed by supernatant removal. The composite was then washed thrice with buffer to remove the free collagen. After the final washing step, 500 µL of Sirius Red solution was added to the tube containing the composite, and the procedure was continued as described in Section 2.6. The results of this experiment indicated that collagen adsorbs onto the surface of PDMS-modified silica composites during incubation. Table 4 presents the experimental outcomes expressed as the quantity (µg) of detected collagen and the percentage recovery, assuming a 100% recovery indicated that the entirety of the collagen (200 µg) present in the incubation mixture at the start was detected in this experiment.

The quantities of detected collagen were in good agreement with the measured decreases in collagen concentration, strongly supporting the hypothesis of collagen adsorption onto the PDMS-modified silica composite. Although the detected collagen amounts represent approximately 88% of the expected values at the respective time points, it is noteworthy that, due to the porous structure of the composite, not all adsorbed particles are accessible to the dye molecules. Significantly, the adsorbed collagen retained its native conformation, enabling interaction with Sirius Red, which is of immense importance in the context of using such composites as collagen carriers, e.g., in guided bone regeneration processes.

**Figure 5:**
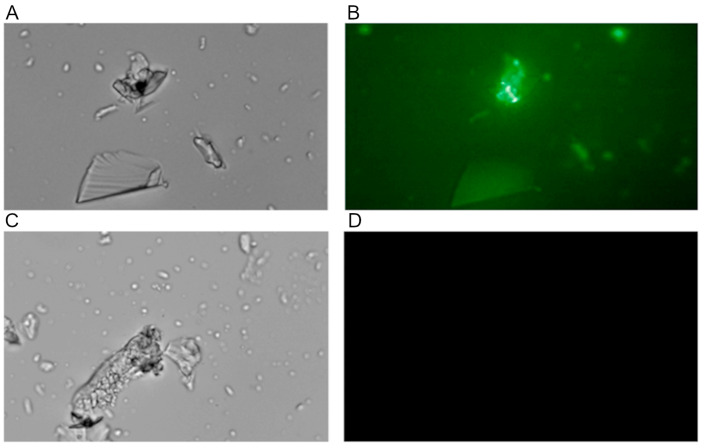
The result of the microscopic observation of a PDMS-modified silica composite incubated with fluorescently labeled collagen (**top** row; (**A**): using a light microscope, (**B**): using a fluorescence microscope) and an unmodified silica composite incubated with fluorescently labeled collagen (**bottom** row; (**C**): using a light microscope, (**D**): using a fluorescence microscope).

The interaction of collagen with the PDMS-modified silica composite was also visualized using fluorescently labeled collagen obtained according to the procedure described in Section 2.8. The observations made while using fluorescence microscopy demonstrated the accumulation of fluorescent protein on the composite surface but only if PDMS was used to modify the composite (Figure 5).

**Figure 6:**
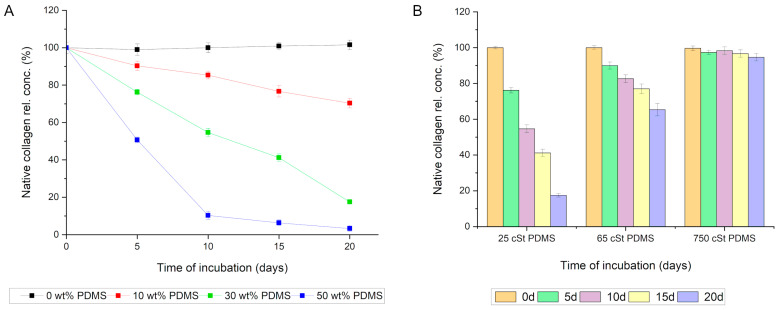
(**A**) Influence of PDMS content (wt%) in the silica composite on the rate of collagen adsorption from a solution; and (**B**) correlation between the viscosity (cSt) of the employed PDMS and the composite’s capacity for collagen adsorption.

In light of the microscopic observation results, an experiment was conducted to determine whether PDMS is responsible for the interaction between the silica composite and collagen. For this purpose, the collagen solution was incubated with four variants of silica composites: (1) devoid of PDMS; (2) containing PDMS at a ratio of 0.1:1 relative to silica (10 wt%); (3) containing PDMS at a ratio of 0.3:1 relative to silica (30 wt%); and (4) containing PDMS at a ratio of 0.5:1 relative to silica (50 wt%). In all the variants, PDMS of 25 cSt viscosity was utilized. The results unequivocally indicate that PDMS is responsible for the interaction with collagen in the composite. The variant without PDMS exhibited no significant statistical capacity for adsorbing collagen. Conversely, even a 10% PDMS content in the composite significantly affected the rate of protein adsorption. Increasing the PDMS content in the composites further accelerated the rate of collagen adsorption and the amount of protein adsorbed (Figure 6A).

Subsequently, the effect of the chain length of the employed PDMS (viscosity) on the capacity of the silica composite to adsorb collagen from the solution was analyzed. It was observed that a lower viscosity of PDMS corresponded to an increased capacity of the composite to adsorb collagen. Among the variants analyzed, the silica composite modified with 25 cSt viscosity PDMS demonstrated the strongest affinity for collagen. Notably, even PDMS with a viscosity of 65 cSt exhibited a significant statistical ability to adsorb collagen from the incubation mixture. Only PDMS with a viscosity of 750 cSt did not show a statistically significant capacity for interactions with collagen (Figure 6B).

**Figure 7:**
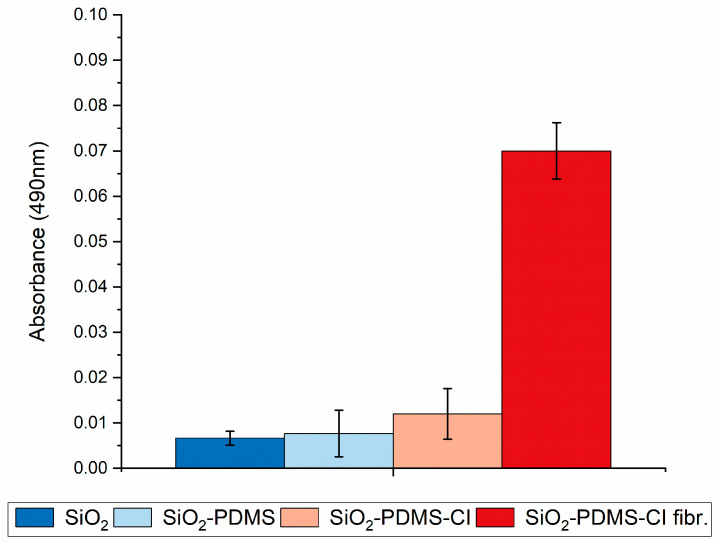
Platelet adhesion confirmed by LDH assay to the following: SiO_2_—pure silica composites; SiO_2_-PDMS—composites modified with PDMS without further incubation with type I collagen; SiO_2_-PDMS-CI—composites modified with PDMS and incubated with native type I collagen; andSiO_2_-PDMS-CI fibr.—composites modified with PDMS, incubated with native type I collagen, and placed in fibril forming conditions.

On the other hand, studies examining the adhesion of platelets to composites using the LDH assay have demonstrated that this phenomenon occurred with statistically significant intensity only in the case of composites containing PDMS that had been previously incubated in a type I collagen solution and then placed in an environment conducive to fibril formation (Figure 7—SiO_2_-PDMS-CI fibr.). In instances where the composite did not contain PDMS or contained PDMS but was not incubated with native collagen, there was no statistically significant interaction with collagen, and, consequently, there were no factors on the composite surface for platelets to adhere to (Figure 7—SiO_2_ and SiO_2_-PDMS, respectively). Similarly, when the composite contained PDMS and was incubated with a collagen solution but the fibril formation process was not induced, only trace amounts of platelets adhered to the composite (Figure 7—SiO_2_-PDMS-CI).

## 4. Discussion

In this study, the Box–Behnken Design was used to model and optimize the parameters of silica composite synthesis. This study examined the impact of H_2_O:TMOS ratio, buffer pH, and PDMS content on the gelation time of the composites. The results showed that the PDMS content did not significantly affect the gelling process. However, the transition from sol to gel was strongly correlated with the H_2_O:TMOS ratio, with higher values resulting in longer gelation times. This is in good agreement with data from other gelation studies [18]. The buffer pH also influenced the gelation of sol, with lower values resulting in longer gelation times. Optimized synthesis parameters were utilized to produce a composite with minimum gelation time and series of composites (without PDMS, with 10%, 30%, and 50% of PDMS modifier). The obtained composites were then subjected to physicochemical tests by ATR-FTIR to examine their composition.

The described study’s results concerning the interaction between native collagen and PDMS-modified silica composites offer significant insights into the material’s biomedical applications, particularly in tissue engineering and drug delivery systems. The observed gradual decrease in native collagen concentration in the solution over 20 days and the statistically significant reduction within the first 5 days of incubation highlight the strong adsorptive interactions between the collagen and the composite material. This finding is in line with studies that demonstrate how surface modifications of silica-based materials can enhance protein adsorption due to an increased surface area and altered surface chemistry [19,20,21].

This experiment’s control samples provide a robust baseline for understanding the specific interaction between collagen and the PDMS-modified silica composite. The lack of a decrease in the native collagen quantity in the control sample without the composite suggests that the observed decrease in the experimental setup was due to the interaction with the PDMS-modified silica and not an inherent instability of the collagen solution over time. This distinction is crucial for confirming the specificity of the interaction and is a methodological strength of this study.

The use of both the modified Lowry method and the Sirius Red dye method for determining the total collagen concentration, including denatured forms, addresses the challenge of accurately measuring protein concentration in the presence of conformational changes. This approach reflects the understanding that different detection methods can yield different results depending on the protein’s condition and a system’s complexity [22].

The finding that collagen retains its native conformation on the PDMS-modified silica composite’s surface is particularly relevant. This suggests that the composite does not denature the protein, which is crucial for maintaining its biological functionality in potential applications like guided bone regeneration or wound healing, where the bioactivity of collagen is a key factor [23]. The results are also supported by the fluorescence microscopy observations, which provided visual confirmation of the protein’s presence and distribution on the composite’s surface.

This study’s exploration of the effect of PDMS content and viscosity on collagen adsorption rates further delves into how the physical and chemical properties of the composite can be tuned to optimize performance. This aspect is particularly relevant in material science and biomedical engineering, where the fine-tuning of material properties can significantly impact the material’s interaction with biological molecules and cells [24].

While there are numerous reports in the literature on the combined use of collagen and PDMS in biomaterials, these studies predominantly focus on the integration of PDMS with collagen, where PDMS serves to impart new mechanical properties to the scaffold. Additionally, the collagen used in such composites often takes the form of fibrils or fibers, mirroring its presence in the human body as molecular scaffolding [25]. However, numerous cells, upon appropriate stimulation, can produce and secrete collagen molecules. A significant achievement of this work is the demonstration that such molecular collagen can interact with silica composites via PDMS, recognized for its high biocompatibility [26]. Furthermore, this collagen remains biologically active, capable of aggregating into higher-order structures, thereby potentially stimulating platelet adhesion and, likely, further aggregation and activation. This process can influence the release profile of substances from the composite or the catalytic activity of enzymes contained within it. Platelet stimulation is one of the initial stages of tissue regeneration processes, such as in bone or skin, and is a desired effect. However, excessive or prolonged stimulation may lead to sustained inflammation. It is noteworthy that the interactions between collagen and the PDMS composite demonstrated in this study require substantial time. This factor could be considered in the design of composites intended as drug carriers, to engineer them in such a way that the active substance is released at a time when a relatively small amount of collagen has been adsorbed.

Molecular dynamics simulations reveal that collagen, characterized by its triple-helix structure, exhibits a stronger affinity for hydrophobic surfaces compared to hydrophilic ones [27]. This preference stems from the hydrophobic interactions between the non-polar amino acid residues in collagen and the PDMS surface, which stabilizes the collagen’s triple helix. The hydrophobic surface of PDMS reduces the thermodynamic penalty associated with the desolvation of these residues upon adsorption, thereby enhancing the structural integrity of the collagen. This interaction is facilitated by the exclusion of water molecules at the collagen–PDMS interface, which lowers the system’s free energy and strengthens the hydrophobic bonds, maintaining the conformational rigidity of the collagen structure. These findings underscore the significance of surface properties in determining the interaction dynamics between collagen and synthetic polymers, with implications for the design of biomaterials in tissue engineering and regenerative medicine.

The interaction between collagen and PDMS-modified silica composites has significant implications for drug delivery, particularly affecting the dissolution kinetics of drugs. The adsorption of proteins such as collagen onto drug carriers can markedly modify the release profiles and bioavailability of therapeutics [28,29]. With PDMS-modified silica composites, the ability to adsorb collagen and potentially other proteins provides a method to modulate the drug release environment. This capability is especially pertinent for therapeutics sensitive to the surrounding protein matrix and for applications necessitating controlled release to specific tissues. By influencing the interface between the drug and its carrier, these interactions can tailor these release kinetics, potentially leading to more effective and targeted drug delivery strategies.

## 5. Conclusions

In this study, we have successfully modeled and optimized the synthesis parameters of silica composites using the Box–Behnken Design. Our findings indicate that the gelation time of these composites, essential for their biomedical applications, is significantly influenced by the H_2_O:TMOS molar ratio and buffer pH but not by the PDMS content. This understanding has enabled us to synthesize composites with varied PDMS contents and examine their physicochemical properties and interactions with native collagen. Our results demonstrate a strong adsorptive interaction between native collagen and PDMS-modified silica composites. This interaction leads to a significant decrease in native collagen concentration in a solution over time, indicating potential applications in tissue engineering and drug delivery systems. Significantly, this study reveals that collagen retains its native conformation on the PDMS-modified silica surface, which is crucial for maintaining its biological functionality. Additionally, the impact of PDMS content and viscosity on collagen adsorption rates offers insights into optimizing composite properties for specific biomedical applications. The interaction between collagen and PDMS-modified silica composites holds substantial implications for drug delivery. The adsorption of proteins like collagen on these composites can significantly alter drug release profiles and bioavailability. This capability to modulate the drug release environment could lead to more effective and targeted drug delivery strategies, especially for therapeutics sensitive to the surrounding protein matrix.

In conclusion, our study not only advances the understanding of the physicochemical properties of silica composites and their interaction with biological molecules but also opens new avenues for the development of advanced biomaterials for diverse biomedical applications.

## Figures and Tables

**Table 1:** Levels of variables and studied responses in the Box–Behnken Design experiment.

Independent Variables	Levels			Response
	−1	0	1	
A–PDMS content (wt%)	0	25	50	Gelation time [min]
B–Buffer pH	3.5	4.5	5.5	
C–H_2_O:TMOS (molar ratio)	10	20	30	

**Table 2:** Analysis of variance (ANOVA) for Box–Behnken Design experiment—gelation time.

Source	SS	df	MS	*F*	*p*	
**Model**	1832.68	3	610.89	275.30	<0.0001	significant
B-Buffer pH	1378.12	1	1378.12	621.05	<0.0001	
C-H_2_O:TMOS	171.12	1	171.12	77.12	<0.0001	
B^2^	283.43	1	283.43	127.73	<0.0001	
**Residual**	28.85	13	2.22			
Lack of Fit	26.05	9	2.89	4.13	0.0925	not significant
Pure Error	2.80	4	0.7000			
**Cor Total**	1861.53	16				

**Table 3:** Summary of the optimized synthesis parameters of the composites.

Sample No.	PDMS Content [% *w*/*w*]	Buffer pH	H_2_O:TMOS [molar ratio]	Predicted Gelation Time [min]	Observed Gelation Time [min]
X1	10.0	5.3	11.0	5.02	5.25
X2	30.0	5.3	11.0	5.02	5.18
X3	50.0	5.3	11.0	5.02	4.96
X4	0.0	5.3	11.0	5.02	4.95

**Table 4:** Quantity of collagen detected on PDMS-modified silica composites.

Time of Incubation (Days)	Quantity of Adsorbed Collagen (µg)	Recovery (%)
0	1.2 ± 0.5	-
5	40.3 ± 3.4	84
10	78.3 ± 4.9	87
15	108.6 ± 5.2	92
20	147.6 ± 8.1	90

## Data Availability

Data are contained within the article.
